# Design, Development, and Testing of an App for Dual-Task Assessment and Training Regarding Cognitive-Motor Interference (CMI-APP) in People With Multiple Sclerosis: Multicenter Pilot Study

**DOI:** 10.2196/15344

**Published:** 2020-04-16

**Authors:** Andrea Tacchino, Renee Veldkamp, Karin Coninx, Jens Brulmans, Steven Palmaers, Päivi Hämäläinen, Mieke D'hooge, Ellen Vanzeir, Alon Kalron, Giampaolo Brichetto, Peter Feys, Ilse Baert

**Affiliations:** 1 Scientific Research Area Italian Multiple Sclerosis Foundation Genoa Italy; 2 Rehabilitation Research Center (REVAL) Faculty of Rehabilitation Sciences Hasselt University Hasselt Belgium; 3 Expertise Centre for Digital Media Faculty of Sciences Hasselt University Hasselt Belgium; 4 Smart ICT PXL University College Hasselt Belgium; 5 Masku Neurological Rehabilitation Centre Masku Finland; 6 National MS Center Melsbroek Melsbroek Belgium; 7 Rehabilitation and MS Center Overpelt Overpelt Belgium; 8 Department of Physical Therapy School of Health Professions, Sackler Faculty of Medicine Tel-Aviv University Tel-Aviv Israel; 9 Sagol School of Neuroscience Tel-Aviv University Tel-Aviv Israel; 10 AISM Rehabilitation Service of Genoa Italian Multiple Sclerosis Society Genoa Italy

**Keywords:** tablet, mobile device, cognitive rehabilitation, cognitive impairment, dual-task training, cognitive-motor interference, dual-task cost, adherence, multiple sclerosis, walking

## Abstract

**Background:**

Dual tasking constitutes a large portion of most activities of daily living; in real-life situations, people need to not only maintain balance and mobility skills, but also perform other cognitive or motor tasks at the same time. Interest toward dual-task training (DTT) is increasing as traditional interventions may not prepare patients to adequately face the challenges of most activities of daily living. These usually involve simultaneous cognitive and motor tasks, and they often show a decline in performance. Cognitive-motor interference (CMI) has been investigated in different neurological populations, but limited evidence is present for people with multiple sclerosis (MS). The use of computerized tools is mandatory to allow the application of more standardized assessment and rehabilitation intervention protocols and easier implementation of multicenter and multilanguage studies.

**Objective:**

To describe the design and development of CMI-APP, an adaptive and interactive technology tablet-based app, and to present the preliminary results of a multicenter pilot study involving people with MS performed in several European centers for evaluating the feasibility of and adherence to a rehabilitation program based on CMI-APP.

**Methods:**

CMI-APP includes user-friendly interfaces for personal data input and management, assessment of CMI, and DTT. A dedicated team developed CMI-APP for Android tablets above API level 14 (version 4.0), using C# as the programming language and Unity and Visual Studio as development tools. Three cognitive assessment tests for working memory, information processing speed, and sustained attention and four motor assessment tests for walking at different difficulty levels were implemented. Dual cognitive-motor tasks were performed by combining single cognitive and motor tasks. CMI-APP implements exercises for DTT involving the following 12 cognitive functions: sustained attention, text comprehension, verbal fluency, auditory discrimination, visual discrimination, working memory, information processing speed, auditory memory, visual memory, verbal analog reasoning, visual analog reasoning, and visual spatial planning, which can be performed during walking or stepping on the spot. Fifteen people with MS (mean age 52.6, SD 8.6 years; mean disease duration 9.4, SD 8.4 years; mean Expanded Disability Status Scale score 3.6, SD 1.1) underwent DTT (20 sessions). Adherence to the rehabilitation program was evaluated according to the percentage of performed sessions, perceived exertion during the training (Borg 15-point Ratings of Perceived Exertion [RPE] Scale), and subjective experience of the training (Intrinsic Motivation Inventory [IMI]).

**Results:**

The adherence rate was 91%. DTT was perceived as “somewhat difficult” (mean RPE Scale score 12.6, SD 1.9). IMI revealed that participants enjoyed the training and felt that it was valuable and, to some extent, important, without feelings of pressure. They felt competent, although they did not always feel they could choose the exercises, probably because the therapist chose the exercises and many exercises had few difficulty levels.

**Conclusions:**

CMI-APP is safe, highly usable, motivating, and well accepted for DTT by people with MS. The findings are fundamental for the preparation of future large-sample studies examining CMI and the effectiveness of DTT interventions with CMI-APP in people with MS.

## Introduction

The conventional approach of physical and cognitive rehabilitation is mainly focused on single-task conditions. Over the past decade, research has devoted increasing attention to dual-task training (DTT) [1] owing to the ascertainment that traditional interventions may not prepare patients for adequately returning to community living (eg, household, school, family, work, and leisure activities). In fact, dual tasking constitutes a large portion of most activities of daily living; in real-life situations, people need to not only maintain balance and mobility skills, but also perform other cognitive or motor tasks at the same time (eg, walking while talking on the phone and rehearsing a shopping list, typing on the smartphone and talking, and preparing meals and talking) [2]. This integrated dual tasking can be defined as the concurrent performance of two tasks that can be executed independently and measured separately and that have distinct goals. This requires adaptive under- or over-additive neural activation in related brain areas [3].

The simultaneous performance of motor and cognitive tasks can be difficult and can lead to worse performance in the motor or cognitive domain or both domains. This cognitive-motor interference (CMI) occurs when performance in a motor or cognitive task decreases on performing a dual task (DT) as compared with performing a single task, which is the so-called dual-task cost (DTC). CMI has been investigated in different neurological populations that usually experience physical and cognitive deficits, including individuals with stroke [4], Parkinson disease [5], and Alzheimer disease [6]. Findings in these populations showed a disproportionate effect of concurrent cognitive tasks on mobility when compared with healthy controls. Moreover, divided attention deficits may prevent neurological individuals from allocating appropriate attentional resources to balance and gait, consequently reducing adaptability to challenging environments (eg, obstacles and uneven paths) and contributing to fall risk [7,8]. Several results indicate that for these neurological conditions, motor and cognitive deficits can be minimized with not only focused single-task training but also targeted DTT [9-11].

Declines during simultaneous performance of cognitive and motor functions are commonly observed in multiple sclerosis (MS) [12,13]. For example, people with MS showed a greatly increased postural sway and a large decrement in variability of anteroposterior and mediolateral sway velocity while executing a simple arithmetic task during balance maintenance as compared with controls [14,15]. Similarly, they showed increased stride time and decreased walking speed during walking under several cognitive conditions (ie, talking) [16-18]. Recently, findings regarding cognitive-motor performances were reported during upper limb tasks [19]. Consolidated evidence of MS rehabilitation regarding walking and cognition (ie, attention, information processing speed, executive function, and long-term memory) [20] independently suggested that the effectiveness on these tasks could be further improved with targeted interventions based on DTT [21]. However, there are still very limited results on the effects of DT rehabilitation strategies in people with MS [22-24], and thus, more clinical and research efforts are required [25]. Standardized research methodology and innovative training programs directed toward meeting the demands of “real-life” situations lack evidence for MS.

Variabilities in task duration, type and complexity of the cognitive task, and training modality (single, consecutive, or integrated dual tasking) limit the availability of standardized testing and training protocols, comparisons across studies, and translation in clinical practice [11]. Therefore, moving toward protocols involving computerized tools is almost mandatory to allow larger sample size inclusion, more standardized protocols for assessment and rehabilitation training, more reliable outcome measures, and easier implementation of multicenter and multilanguage studies.

Moreover, to date, most technological solutions on the market are able to provide multisensory feedback and modulate exercise complexity according to the patient’s capacity. However, rarely, they implement tests and exercises suited and tested for neurological rehabilitative interventions, as well as appropriate interfaces to plan assessment and training in both the cognitive and motor domains independently or together. Moreover, the high costs and lack of portability severely limit their use in clinical practice. For these reasons, the increasing availability of portable devices with adequate memory and calculus performance prompted us to develop a new solution for DT investigations based on a tablet app.

Owing to these considerations, a mobile tablet-based app was proposed, designed, and developed to assess CMI, deliver DT exercises, and investigate DTT effects in people with MS. It allows the application and objective quantification of standardized assessment and rehabilitation interventions, as such opening the “black box” of rehabilitation content.

In this paper, we describe the design and development of CMI-APP, an adaptive and interactive technology tablet-based app, as well as the results of a multicenter pilot study involving people with MS that was performed in several European centers to evaluate the feasibility of and adherence to a rehabilitation program based on CMI-APP, the perceived exertion during the training, and the subjective experience regarding the training. This is fundamental in preparation for future large-sample studies examining CMI and the effectiveness of DTT interventions with CMI-APP in people with MS.

## Methods

### Study Centers

The participating centers were as follows: Rehabilitation Research Center (REVAL) and Expertise Centre for Digital Media of Hasselt University in Belgium; Italian Multiple Sclerosis Society (AISM) Rehabilitation Service of Genoa and Foundation of AISM (FISM) in Italy; Smart ICT of the PXL University College of Hasselt in Belgium; Rehabilitation and MS Center Overpelt in Belgium; National Multiple Sclerosis Center Melsbroek in Belgium; Masku Neurological Rehabilitation Centre in Finland; AZ Klina, campus De Mick, rehabilitation, Brasschaat in Belgium; Centre Hospitalier Universitaire de Liège in Belgium; and Multiple Sclerosis Center, Sheba Medical Center, Tel-Hashomer in Israel. The study was approved by the Ethics Committee of CHU Liège, Belgium, as well as the local ethics committee of each participating center.

The Expertise Centre for Digital Media of Hasselt University and Smart ICT of the PXL University College of Hasselt developed CMI-APP in collaboration with REVAL. The Centre Hospitalier Universitaire de Liège and Masku Neurological Rehabilitation Centre were involved in the test-retest reliability study of the assessment module of CMI-APP [18]. In addition, therapists from the MS rehabilitation centers in Belgium were involved in the development part of the study. For the multicenter pilot study, five centers recruited people with MS. These centers were AISM; Multiple Sclerosis Center, Sheba Medical Center; National Multiple Sclerosis Center Melsbroek; AZ Klina, campus De Mick; and Rehabilitation and MS Center Overpelt.

Based on clinical experience and the knowledge of researchers and therapists, the types and difficulties of cognitive and motor exercises were discussed during several meetings. If there was uncertainty about the duration of an exercise, a literature search was performed and the approach was tried out in clinical practice. The responses of participants to the Dual Task Questionnaire of Evans et al and their advice in the test-retest reliability study [18,26], as well as the long experience of working with people with MS helped us to identify the needs and appropriate exercises.

### Development of CMI-APP

#### Overview

The initial design and development of CMI-APP was started in 2015, with start-up support from the Flemish Multiple Sclerosis Society. The concept of the app was discussed in a group of rehabilitation scientists and clinicians with MS expertise (ie, physiotherapy and neuropsychology), people with MS, and computer scientists from the Expertise Centre for Digital Media of Hasselt University (Belgium) and Smart ICT of PXL University College (Belgium). The multidisciplinary team regularly met and collaborated by using essential techniques of user-centered design and development, such as iterative development and evaluation of intermediate prototypes. The core development period was from January to December 2016, when the version of the app used in the study was finalized. In the second step, cultural adaptations and translations to other languages of the partners involved in the project (Italian, Hebrew, Finnish, and French beside Dutch and English) were performed to prepare for an international multicenter approach. This was supported by the European network for MS rehabilitation, Rehabilitation in Multiple Sclerosis, and Swedish PROMOBILIA foundation. In particular, in order to maintain consistency among the partner languages, the development team, information technology specialist, and representative of each of the partners continuously interacted for accurate translation and adaption of the text on the objects of the graphical user interface (GUI) (eg, labels and buttons) and for the production of the auditory files used in CMI-APP. To allow more flexibility, the app was designed to be easily extended to other languages.

CMI-APP has been developed for any Android tablet above API level 14 (version 4.0), using C# (Microsoft Corp, Redmond, Washington, USA) as the programming language and Unity and Visual Studio (Microsoft Corp) as development tools. These common platforms facilitate accessibility for the centers and therapists involved in the study and are good choices for possible further development and deployment of the app in rehabilitation practice after the study.

The GUI of CMI-APP was implemented through three different but related modules. The main menu (Multimedia Appendix 1) allows the therapist to add new patients or therapists and to retrieve previously created people for assessment and training. Patients and therapists are added with unique codes (ie, “Patient code” and “Therapist code”). Additional information about the selected patient (eg, visual problems) and the current session (eg, bad sleep) can be added as a note. Furthermore, two numerical text boxes are provided to add the baseline number of steps in a predefined temporal range (eg, 1 minute) during walking and stepping on the spot, which are both assessed at the first evaluation (eg, through a pedometer). The main menu also allows language selection (currently, Dutch, Italian, French, Hebrew, and Finnish). Finally, there are two buttons “Start assessment” and “Start exercises” to access the modules for assessment and training, respectively.

#### Assessment Module

Assessment tests for three different cognitive functions (working memory, information processing speed, and sustained attention) at various difficulty levels are implemented in CMI-APP. These are “Titrated digit span backwards,” “Auditory vigilance with alphabets,” and “Serial counting backwards by 7.” In “Menu–Assessment,” these three types of tests are available for selection to be performed on their own or in combination with motor tasks. Cognitive tasks were chosen considering that working memory, information processing speed, and attention are among the most affected cognitive domains in MS and considering the results of pilot studies and feasibility during walking [18,27]. Currently, according to clinical and experimental experience, the following four common walking activities, which are carried out in daily life but differ in motor complexity and require attention or adaptation, are included in the testing protocol: walking at a self-selected speed, walking at a self-selected speed while carrying a cup filled with water, walking at a self-selected speed while stepping over various obstacles (eg, 10-cm height, 10-cm width, soft material, and every 3 m in a straight line), and walking crisscross at a self-selected speed from one cone to another (eg, every 2 m with a fixed 80-cm width in between). The motor tasks (actual single or dual motor tasks) were chosen according to the findings of previous studies investigating reliability in persons with neurological conditions during various walking tasks [18,27-31]. It is suggested to perform walking on a 30-m quiet walkway that is free of obstacles and has marked start and turning lines (eg, 80 cm). Before the execution of these tasks, the walking activities should be tried in order to perform them without uncertainty. For all motor tasks, the therapist should demonstrate how to walk over the walkway, and participants should try to walk on a part of the walkway. The different complexities of motor tasks allow for personalization of the difficulty level depending on the individual’s ability, the need to train for specific problems, and the disease progression. Thus, the performance of a patient can be assessed under a total of 19 conditions (three single cognitive conditions, four single or dual motor conditions, and 12 dual cognitive-motor conditions) (Multimedia Appendix 2). Each test lasts for 60 seconds, and the result is stored only if it is successfully performed. For safety, it is suggested for the examiner to walk close to but behind the participant. Moreover, it is suggested to put the tablet in a case with a sling wearable over the shoulder, so that, if needed, the therapist can drop the tablet (it will hang on the therapist’s neck) without damaging it and catch the patient.

The order in which the blocks of single cognitive, single or dual motor, or dual cognitive-motor tasks are presented, as well as the sequence of each separate task within one block is optimally randomized. To make the assessment easier and more reliable at different time points, the order automatically remains the same for the patient. Multiple conditions should be evaluated for a complete assessment of CMI. In fact, usually, DT performance is assessed through one DT condition/paradigm, which is mostly quantified as motor DTC. DTC may suggest whether and how attention resources, executive functions, and working memory affect a motor task (ie, motor DTC) or whether and how walking affects cognitive tasks (ie, cognitive DTC) [32-35]. Considering that different cognitive or motor tasks compete for cognitive or motor resources to varying extents, using only one cognitive or motor task may not be sufficient to explain CMI in its entirety [18].

Nevertheless, a therapist can decide to administer only a reduced subset of conditions (cognitive, motor, or cognitive-motor). Descriptions of the cognitive tasks are provided below.

##### Titrated Digit Span Backwards

This mental tracking task requires sustained attention, working memory, and information processing speed. Patients listen to a titrated string of digits (eg, 3-2-5-7-9), which is presented at a rate of one per second, as commonly used in standard neuropsychological tests [36]. The digit order in the string is automatically and randomly generated by an app routine that follows ordered sampling without replacement for the digits 1-9 (eg, for a string length of three digits, the number of orders is 9×8×7=504).

Subsequently, they are requested to repeat the string in reverse order. Before the test, the therapist can define a personalized sequence length with the procedure activated through the button “Assess span length” (Multimedia Appendix 3). Four trials are performed at each sequence length starting from a length of three digits. If three out of four trials at a given length are correct, the patient is considered to have passed the test for that specific sequence length, and the length is increased by one digit. Each patient’s digit span length is determined as the largest sequence length for which the patient succeeds in at least three out of four trials. The interface of the digit span test is similar to that of the determination of the titrated span length and is activated through the button “Start the exercise” (Multimedia Appendix 3). Before starting the test, the therapist can ultimately set the digit span length of the patient. After the therapist types the digits responded by the subject and pushes the button “Enter,” the next sequence is delivered.

##### Auditory Vigilance With Alphabets

This reaction time task requires processing speed, with detection of underlying attention deficits. In this test, patients listen to 60 seconds of recorded letters at the presented rate of one letter per 2.5 seconds and have to say aloud “yes” every time they hear the two target letters indicated before starting the trial (a total of 24 letters, of which 10 are target letters). Target letters were chosen as not very common or very rare in everyday speech and not easily confused with other letters (each country has its own version based on some common rules). Each time, the order of 24 letters is automatically and randomly generated by an app routine that firstly extracts a combination with replacement of 14 sequence nontarget letters (ie, 24 sequence letters − 10 sequence target letters) from 24 alphabet nontarget letters (ie, 26 alphabet letters − 2 alphabet target letters) and secondly randomly combines the 14 extracted nontarget letters with the 10 target letters.

The therapist only has to push the button “YES” when the participants says “yes.” False positive (wrong answers) and negative answers (omissions) are automatically counted as incorrect (Multimedia Appendix 4).

##### Serial Counting Backwards by 7

This mental tracking task requires sustained attention and information processing speed. In this test, patients have to consecutively subtract 7 starting from a given number (different numbers at each measurement time point; the starting number is automatically and randomly selected by an app routine in the range 101-199). For example, take 7 away from 101 (value 94), take 7 away from 94 (value 87), and so on. However, if the patient makes a mistake, but he/she correctly goes on from it (eg, 101, 95, 88, and so on), it is only counted as one mistake, and subsequent numbers are considered accurate. The therapist has to type the responded number and push “Enter” to save it (Multimedia Appendix 5). The number of correct subtractions is automatically counted.

#### Training Module

CMI-APP offers easy access to DTT interventions for people with MS and therapists, as it is conceptualized in such a way that cognitive exercises are combinable with selected motor tasks.

**Figure 1:**
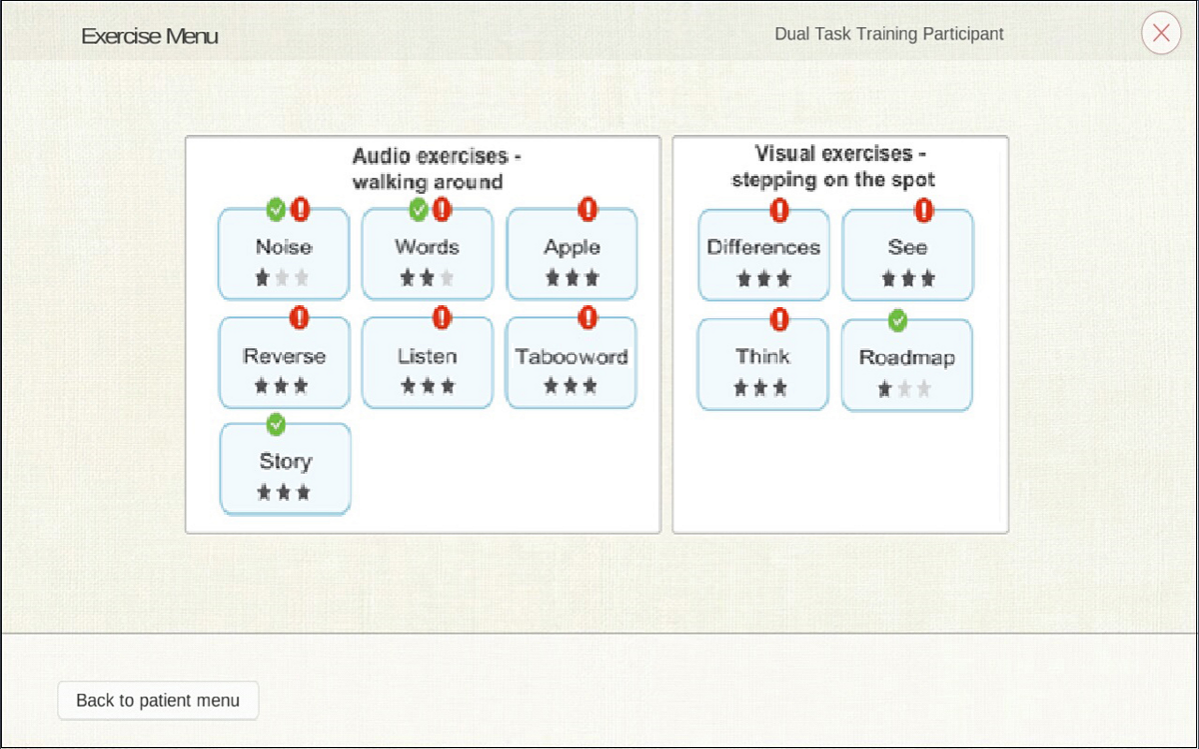
Selection and start of dual-task training. The interface is split into two parts as follows: audio exercises mainly executable by walking around (ie, “Apple,” “Listen,” “Noise,” “Reverse,” “Story,” “Tabooword,” and “Words”) and visual exercises mainly executable by stepping on the spot (ie, “Differences,” “Road map,” “See,” and “Think”). The number of dark stars indicates the exercise difficulty level (three stars indicate level 3). When the exercise is performed in the current session, it is marked with a green check mark. When the exercise is performed in the previous session, it is marked with a red exclamation mark.

Training exercises for 12 different cognitive functions (sustained attention, text comprehension, verbal fluency, auditory discrimination, visual discrimination, working memory, information processing speed, auditory memory, visual memory, verbal analog reasoning, visual analog reasoning, and visual spatial planning) are implemented in CMI-APP. Detailed descriptions of the 11 exercises can be found in Table 1. “Noise,” “Words,” “Apple,” “Reverse,” “Listen,” “Tabooword,” and “Story” can be performed while walking, whereas “Differences,” “See,” “Think,” and “Roadmap” are better suited during stepping on the spot (Figure 1).

**Table 1:** Cognitive exercises implemented in CMI-APP.

Exercise type		Cognitive function	Description
**Exercises with auditory stimuli while walking**			
	Noise	Auditory discrimination	Recognizing two to four target noises over different sounds/noises.
	Words	Working memory and information processing speed	After hearing a given word, formulating a new word with the first, last, second, or fourth letter of the given word.
	Apple	Sustained attention	Reaction to one or two target word(s) over semantically equal or semantically different words.
	Reverse	Working memory and information processing speed	After hearing a given word of three to seven or more letters, spelling the word in reverse (letter by letter).
	Listen	Auditory memory	Each time a new word in a list of words is heard, saying if the word was already heard or not
	Tabooword	Verbal fluency	Describing a target word (without using one or three forbidden taboo words) while following some rules.
	Story	Text comprehension	After hearing a story, responding to three multiple choice questions about the story.
**Exercises with visual stimuli while stepping on the spot**			
	Differences	Visual discrimination	While seeing two images, saying if the images are the same or different in a given time.
	See	Visual memory	After seeing a smiley, saying which smiley is just seen among three presented.
	Think	Verbal and visual analog reasoning	Making associations between pictures, solving assignments, and completing logical sequences.
	Roadmap	Visual spatial planning	After seeing a roadmap with locations, roundabouts, houses, and trees, at each intersection, saying which direction to go to reach the given destination.

As examples, we present the following three exercises below: “Tabooword” for verbal fluency, “Differences” for visual discrimination, and “Roadmap” for visual spatial planning.

##### Tabooword

In this exercise, the patient hears a word (ie, the guess word) that he/she then has to describe to the therapist. However, the patient has to follow some rules and specifically cannot (1) use the word itself or parts of the word; (2) use words that are derived from the word; (3) use gestures and noises; (4) use abbreviations, initials, or clues like “sounds like” and “rhymes with;” and (4) use the taboo words indicated before exercise start. For example, if the guess word is “apple” and taboo words are “fruit,” “red,” and “core,” the possible solutions are “eat healthy,” “snow white ate an,” and “Jonagold, Granny Smith.”

**Figure 2:**
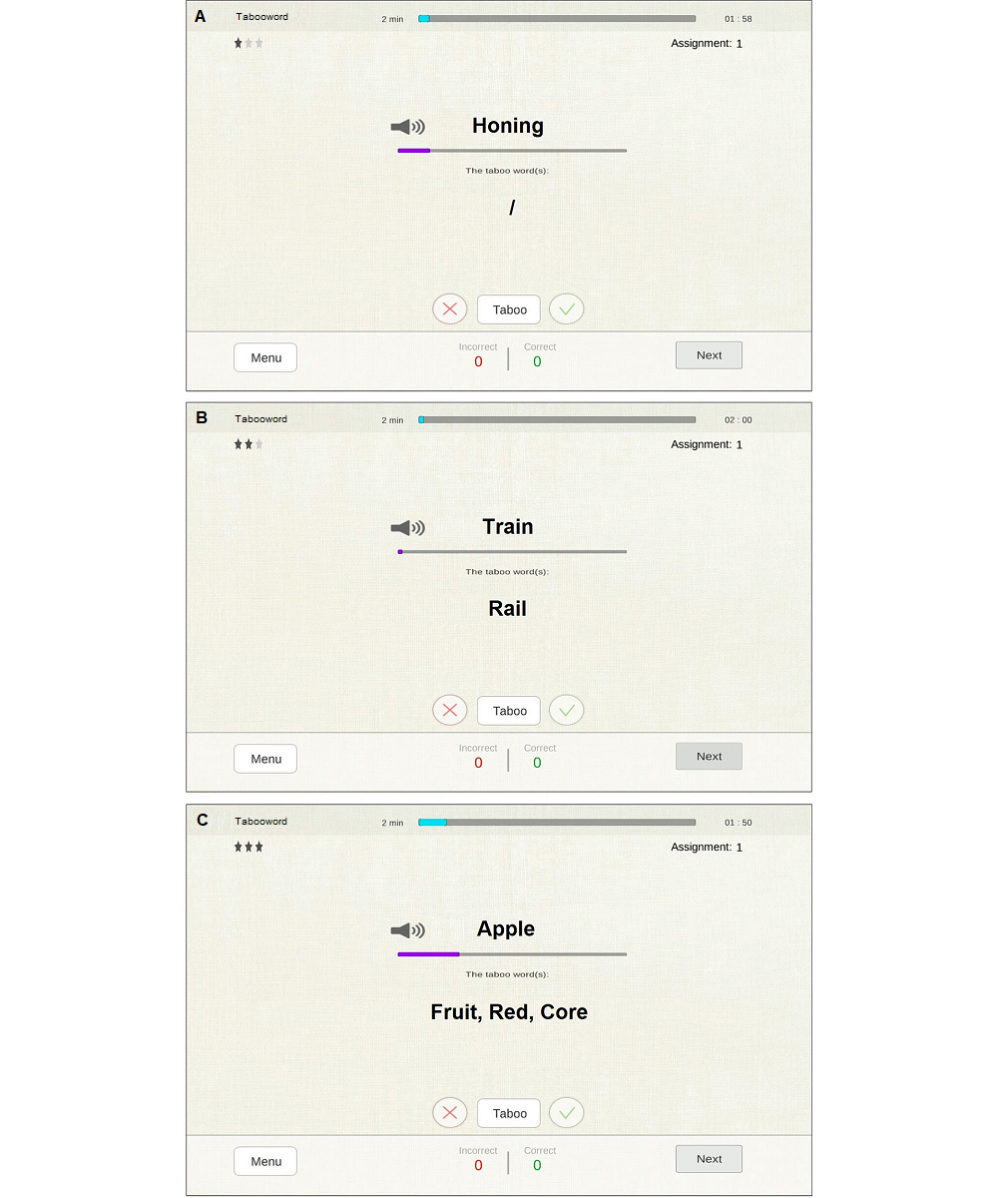
Tabooword exercise. (A) Low difficulty level (20 seconds for description, no taboo words); (B) medium level (30 seconds for description, one taboo word); (C) high level (40 seconds for description, three taboo words).

At the low difficulty level, the patient has to describe a word in 20 seconds and there are no taboo words. At the medium level, the patient has to take into account only one taboo word and the description is required in 30 seconds. At the high level, three taboo words are presented and the test lasts 40 seconds (Figure 2).

The therapist judges the clarity of the description by pressing score buttons as follows: green for a good description within the time limit and red for a bad description. The “Tabooword” button has to be pressed when the patient breaks one of the five rules (eg, uses the word apple to describe an apple tree). The “Tabooword” button can be used three times before the full assignment is scored as wrong; thus, each time the “Tabooword” button is used, the patient receives minus one-third of the score. When the therapist is satisfied with the score before the time limit is reached, the next assignment can be manually provided. Feedback on the marked mistakes can be delivered before advancing to the next assignment. When the timer runs out, the assignment is automatically scored as incorrect. When the patient wants to skip the assignment without trying, the therapist can press the “Next” button without using the score buttons.

##### Differences

**Figure 3:**
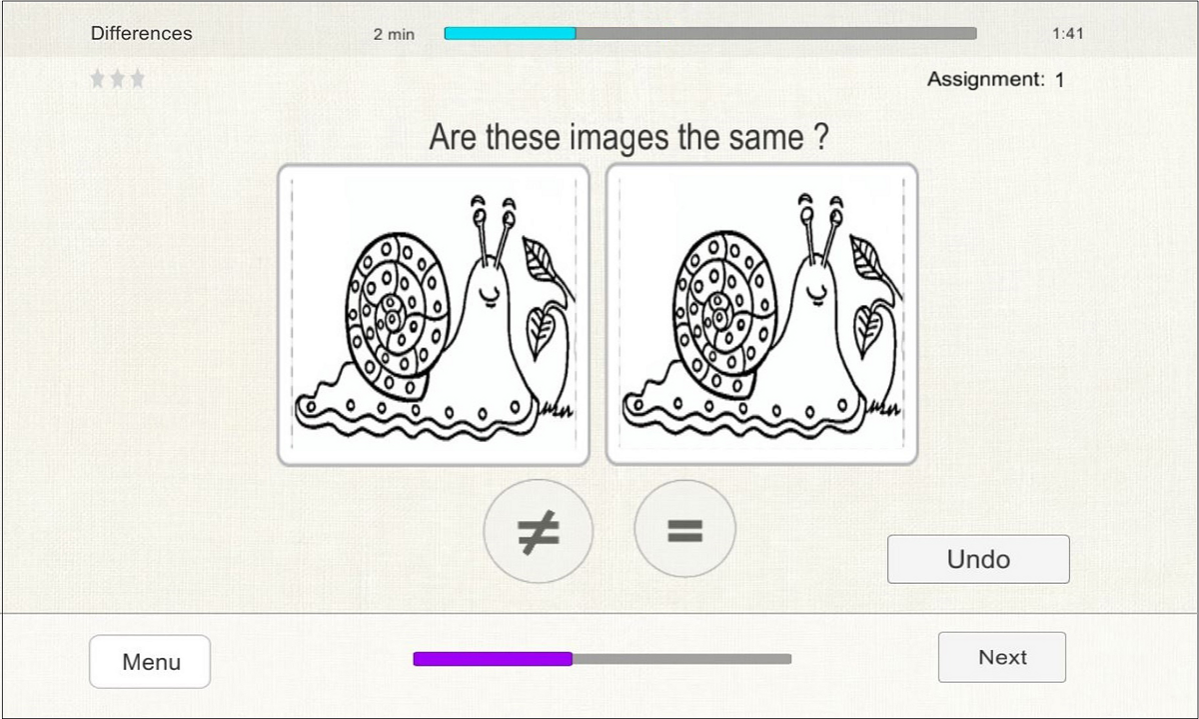
Differences exercise. Two pictures of the high difficulty level are matched.

In this exercise, the patient is shown two pictures and has to tell the therapist whether these pictures are the same or different (Figure 3). Specifically, at the low difficulty level, the patient has 15 seconds to judge the equality and pictures contain more than one difference. At the medium level, the patient has 20 seconds to judge the equality and pictures contain only one difference. At the high level, the patient has 30 seconds to judge the equality and pictures contain one small difference. The patient does not need to refer to the differences and needs to only state whether the pictures are the same or different. If the patient answers “different,” the therapist has to press the button with the different mark (≠). If the patient answers “same,” the therapist has to press the button with the same mark (=). After entering the answer, the “≠” and “=” buttons disappear and the therapist has to proceed to the next assignment by pressing the button “Next.” The same button can be used if the patient wants to skip the current assignment. If time runs out, the assignment is marked as incorrect.

##### Roadmap

**Figure 4:**
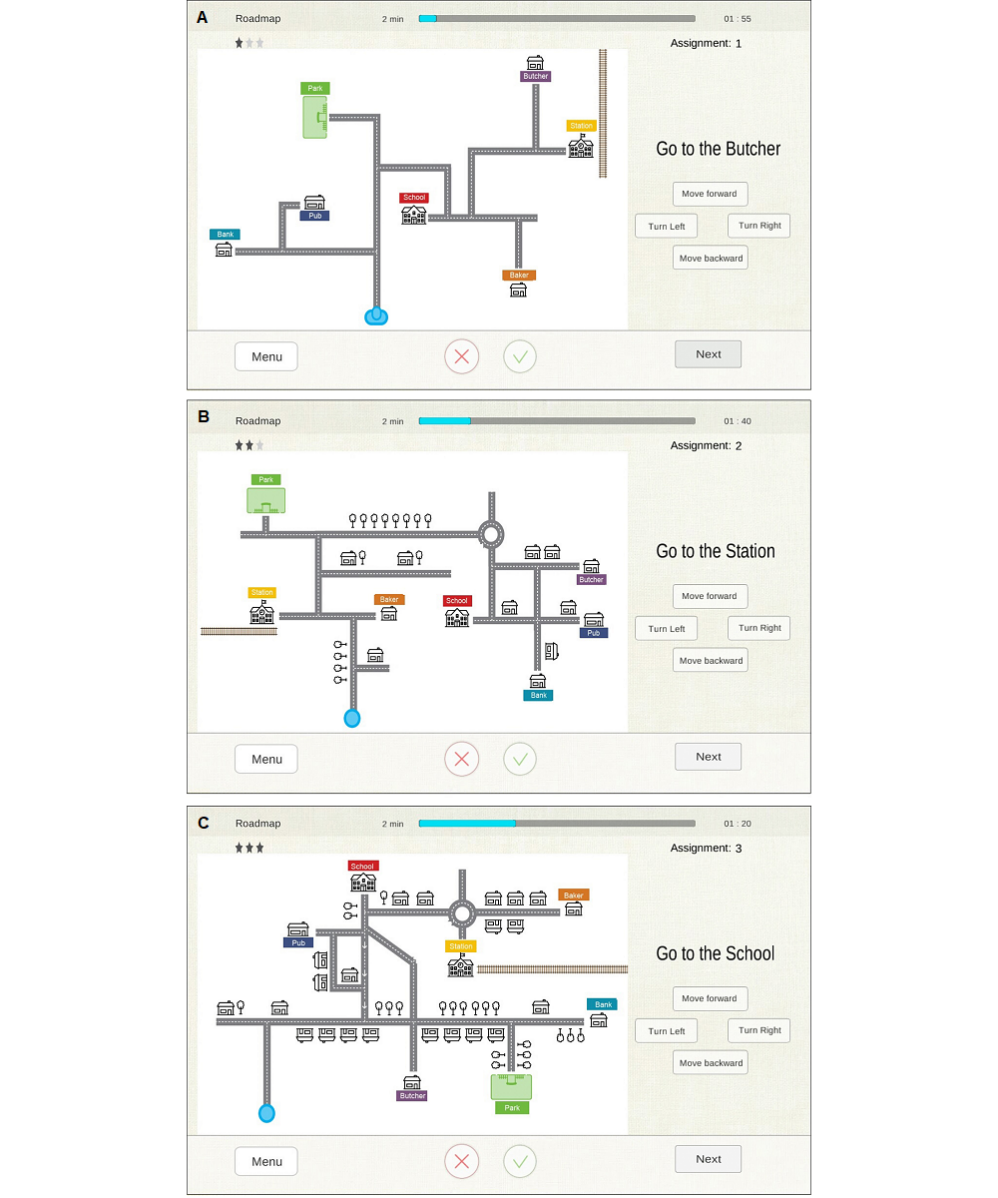
Roadmap exercise. (A) Low difficulty level (without map distractors and with a head as the blue icon to help with orientation); (B) medium level (with roundabouts, houses, and trees as distractors on the map and a dot as the blue icon [invisible person’s orientation]); (C) high level (with roundabouts, houses, trees, and one-way streets as distractors on the map and a dot as the blue icon [invisible person’s orientation]).

In this exercise, the patient is shown a roadmap with seven locations (eg, butcher, park, and school). The current location of the patient is indicated by a blue icon (person symbol) (Figure 4). On the right side, the patient can see the destination of the route (eg “Go to the butcher”). The patient should tell the therapist at each intersection which direction he/she wants to go (ie, forward, backwards, to the left, or to the right) in order to move in the direction of the destination. The therapist has to enter this on the tablet, and the patient’s location will change on the map. The patient has to continuously pay attention to the orientation in order to correctly indicate the direction in which he/she wants to go. The low difficulty level is implemented without map distractors and with a head as the indicator (blue icon) to help with orientation. The medium level involves roundabouts, houses, and trees as distractors on the map and a dot as the indicator (blue icon; invisible person’s orientation). The high level involves roundabouts, houses, trees, and one-way streets as distractors on the map and a dot as the indicator (blue icon; invisible person’s orientation).

The patient reads the assignment/target location and starts navigating by telling the therapist which direction he/she wants to go on the map. The therapist enters the patient’s direction by pressing the corresponding button. The therapist does not need to remember or compensate for the orientation of the patient on the map. However, when the patient states a direction, he/she has to take into account his/her own mental orientation. For this reason, in order to better stimulate visual spatial planning, it is suggested to not allow changes in physical orientation. When the patient reaches the target location, considers the assignment complete, or wants to stop the assignment, the therapist scores the assignment by using the score buttons. The red cross indicates that the patient failed the assignment (ie, standing at the wrong location or making mistakes during the route). The green mark indicates that the patient passed the assignment (ie, correct location and correct route). After entering a score, the score buttons disappear.

#### Feedback

**Figure 5:**
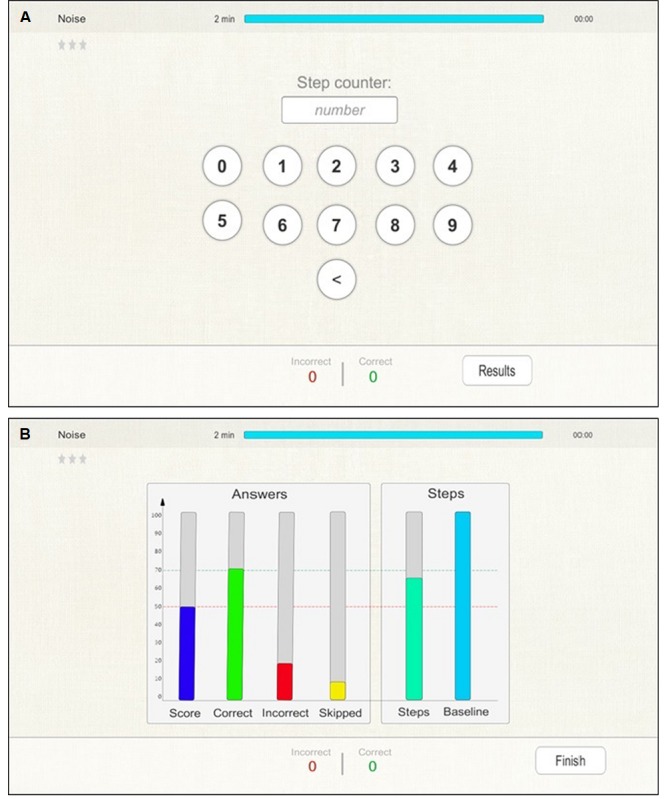
Feedback exercise. (A) The interface to enter the number of steps and the “Results” button to continue to the visual overview of the results as shown in (B). (B) Six colored bars represent the percentage composite score of correct, incorrect, and skipped answers; the percentages of correct, incorrect, and skipped answers for the given assignments; and the percentages of the steps per minute during the exercise and at baseline. A red line indicates a percentage performance of 50%, and values below 50% for cognitive score and steps are considered for level down in the next session. A green line indicates a percentage performance of 70%, and values above 70% for cognitive score and steps are considered for level up in the next session.

At the end of each exercise, the therapist is obligated to enter the number of steps counted during walking or during stepping on the spot (Figure 5). In order to make counting of the steps and training administration feasible, a pedometer was used. After entering the number of steps, the “Results” button allows to continue to the visual overview of the results from the current exercise. The interface offers feedback on the cognitive and motor performances during dual tasking. In particular, the cognitive performance is displayed with colored bars; the first representing a percentage composite score calculated according to the numbers of correct, incorrect, and skipped answers, and the others representing the percentages of correct, incorrect, and skipped answers for the given assignments. Two more colored bars represent the percentages of the steps per minute during the exercise and at baseline. It should be taken into account that if the number of steps is smaller than at baseline, the baseline bar is 100% and the steps bar is the percentage of steps with reference to the baseline. On the other hand, if the number of steps is greater than at baseline, the steps bar is 100% and the baseline bar is the percentage of steps per minute during the exercise. To provide more complete information to the therapist, the absolute values of the correct and incorrect answers during the exercise are displayed at the bottom. A red line indicates a percentage performance of 50%, and values below 50% for cognitive score and steps are considered for level down in the next session. A green line indicates a percentage performance of 70%, and values above 70% for cognitive score and steps are considered for level up in the next session (Figure 5).

A visual overview of the results could be displayed to the patient if it is considered a valuable way to stimulate his/her cognitive and physical performances.

#### Routines for Auditory Files, Logs, and Data Storage

Auditory files (ie .wav format) are delivered by customized routines. During training exercises, if the setup includes the use of a wireless headset microphone (eg, Logitech H800 USB Wireless Headset with Noise Cancelling Microphone; Logitech, Lausanne, Switzerland), the patient’s responses can be recorded. Similarly, during assessment, logs with assignments, patient’s answers, response accuracy, and .wav files were recorded. The audio recording could be used to later calculate the percentage of correct answers in case of suspicion of typographical errors by the therapist. Data recorded during the CMI assessment and DTT were sent via wireless service from each center to a central server in order to store data for subsequent analyses.

#### Instruction Booklet

Although the cognitive tasks implemented in CMI-APP could be useful for both outpatient and at-home cognitive rehabilitation, DTT involving walking and stepping on the spot (to be safe for neurological conditions) should be performed in a clinical setting and delivered by a trained therapist. For this reason, to make the use of CMI-APP more practical, safer, and widespread, we provide an instruction booklet for assessment and training. Owing to the user-friendly and tailor-made interface of CMI-APP and the easiness for learning how the cognitive, motor, and cognitive-motor tasks have to be executed, the booklet is a valid alternative to a training course for therapists. In particular, the booklet shows the screens encountered in CMI-APP with annotations and instructions necessary to complete each screen for each assessment or training exercise.

### Study design

#### Overview

In order to evaluate the feasibility and adherence to a rehabilitation program based on CMI-APP, perceived exertion during the training, and subjective experience regarding the training and the training activities, a pilot test was performed in people with MS. The information presented here is a small part of a larger study on the assessment and DTT of CMI in people with MS. The whole study consisted of an assessment study with a test-retest design and an intervention study as described previously [18,37]. The intervention study was a multicenter, randomized, two-arm, controlled trial consisting of the integrated DTT as provided by the CMI-APP and a single mobility training group. The results of this study on DT and cognitive and motor performances are published in another manuscript [37]. Here, the development and technical details of the CMI-APP are described. Furthermore, a subsample of the intervention study sample receiving DTT was analyzed for adherence, perceived exertion, and subjective experience regarding the training, and the data are presented.

#### Patients

A total of 15 people with MS (10 women and five men) were recruited from the participating centers (five from Belgium, seven from Italy, and three from Israel). The inclusion criteria were as follows: (1) diagnosis of MS according to McDonald criteria [38]; (2) all types of MS; (3) age between 18 and 65 years; (4) Expanded Disability Status Scale (EDSS) [39] score ≥2 and ≤5, as determined by neurologists or trained clinicians, which includes ability to walk without a waking aid or rest for 200 m; (5) no relapse within the last 30 days; (6) no changes in disease-modifying treatment and no corticoid therapy within the last 50 days; and (7) appropriate cognitive capacity measured by the Mini-Mental State Examination (MMSE) (score ≥26) [40]. The exclusion criteria were as follows: (1) other medical conditions interfering with mobility (eg, acute/subacute fractures and pregnancy >20 weeks); (2) other neurological diagnoses (eg, stroke and Parkinson disease) or MS-like syndromes (eg, neuromyelitis optica); (3) inability to understand and execute simple instructions; (4) problems with hearing or vision interfering with assessment or training (even after adjustment with hearing aids or glasses); and (5) ongoing DT training, other interfering physical therapy, or cognitive training/neuropsychological rehabilitation (eg, balance and walking rehabilitation, occupational therapy, cognitive rehabilitation, and speech rehabilitation).

All participants in this pilot study provided informed consent. The study was conducted in accordance with the Declaration of Helsinki (1964) [41].

#### Training Program

The participants performed outpatient DTT of 20 sessions, with a frequency of five times over 2 weeks (two or three times in the first week and three or two times in the second week) during 8 weeks. Each session lasted 45 to 60 minutes with a total DTT time of 30 minutes. DTT consisted of the execution of the cognitive exercises implemented in CMI-APP (Table 1) while participants walked or stepped on the spot. Training was performed in a dedicated rehabilitation room and supervised by a therapist specifically trained to tailor make the cognitive exercises with difficulty progression according to feedback performance of the previous training session, safety, quality consideration, and patient preference regarding the exercises. Progression to a higher difficulty level was allowed if the number of steps per minute during the exercise was ≥70% of baseline and if the accuracy of answers in the exercise was ≥70%. Moreover, the therapist decided the level increment if the safety and quality judgements of participant performance were respected. Regression to a lower difficulty level occurred if the number of steps per minute during the exercise was <50% of baseline or the accuracy of answers in the exercise was <50%, or for safety/quality reasons according to therapist judgement. The therapist counted the steps using a simple user-friendly pedometer (SW200 Digi-Walker Pedometer, Yamax, Bridgnorth, UK), which could be compared to those recorded at the first evaluation.

For each exercise, participants always started from the first level provided by CMI-APP. If the performance met the criteria for progression, the second level was adopted. Participants were advanced to the third level or downgraded to the first level if the exercise performance was good (advanced) or bad (downgraded) thrice (not necessary consecutively). To avoid overloading the participants, only five out of 12 cognitive functions while walking at usual pace or stepping on the spot were trained per session (eg, working memory, information processing speed, auditory memory, visual discrimination, and visual memory trained with four exercises, such as “Words,” “Listen,” “Differences,” and “See”). Subjects were instructed and encouraged to perform both tasks as good as possible and were free to prioritize. This might allow people to decide unconsciously which task to prioritize, as in everyday life. This aspect was considered in line with the study by Silsupadol et al [42] showing that in elderly adults, variable-priority training (ie, no instructions to prioritize either the motor or cognitive task) was more effective for improvement in mobility or cognitive outcomes under DT conditions than fixed-priority training (ie, instructions to prioritize either the motor or cognitive task).

#### Outcomes

Adherence is the extent to which the number of training sessions completed by the patient corresponds with the number of sessions of DTT. The patients were involved in a single-arm pilot study, and they could choose to not execute the sessions and eventually drop out. Any adverse effects (eg, falling and pain) during the training period were also recorded.

After each training session, perceived exertion during the training was assessed using the Borg 15-point Ratings of Perceived Exertion (RPE) scale [43]. The scale score ranges from 6 to 20, where 6 indicates “no exertion at all (rest)” and 20 indicates “maximal exertion.” It should reflect how heavy or strenuous the exercise was according to the patient, combining all sensations and feelings of physical stress, effort, and fatigue.

After the 20 sessions of the training program, a 30-item questionnaire, the Intrinsic Motivation Inventory (IMI) [44], was administered to assess the patient’s subjective experience regarding the trained activities. This instrument, through several subscales, assesses the participant’s interest/enjoyment, perceived competence, effort/importance, felt pressure and tension, value/usefulness, and perceived choice while performing the training. The possible answers in the multiple-choice questions ranged from 1 (“not at all true”) to 7 (“very true”). However, several reverse items were present. For these items, the response score was subtracted from 8, and the result was used as the item score. The items of each subscale were interest/enjoyment (score range 5-35); perceived competence (score range 5-35); effort/importance (score range 5-35); pressure/tension (score range 5-35); value/usefulness (score range 6-42); and perceived choice (score range 4-28). The subscale scores were obtained by summing the scores of the items of each subscale; higher scores indicated positive subjective experience.

Moreover, at the end of the training, an open-ended survey on training perception was administered to both the patient and therapist. The patient and therapist had to refer explicitly to what were the strong and weak aspects of the proposed training, and patients were asked if they prioritized tasks during the exercises.

## Results

The mean age of the patients was 52.6 years (SD 8.6, range 34.9-63.7), and the mean disease duration was 9.4 years (SD 8.4, range 0.8-25.1). Among the 15 patients, nine had a relapsing-remitting form of MS and six had a progressive form. The mean EDSS score was 3.6 (SD 1.1, range 2-5). Among the participants, 40% (6/15) had a bachelor’s or master’s education level, 27% (4/15) had a tertiary education level, and 33% (5/15) had an upper secondary or lower education level. The mean body mass index was 26.2 (SD 4.3). All patients showed appropriate cognitive capacity (mean MMSE score 28.7, SD 1.3; range 26-30), although they reported presence of DT interference (mean DT screening list 4.7, SD 2.7; range 0-9).

Most patients performed 20 training sessions (median 20, IQR 16-20), with a mean adherence of 91% (18.1/20). No adverse effects of the DTT were reported in any of the participants. On average, participants perceived the DTT as “somewhat hard” as shown by a mean RPE score of approximately 13 (mean 12.6, SD 1.9; range 8.8-16.1).

As recorded with the IMI, participants in general enjoyed the training (IMI interest/enjoyment: mean 27.5, SD 5.1) and felt that it was valuable and, to some extent, important (IMI value/usefulness: mean 31.1, SD 9.5; IMI effort/importance: mean 23.5, SD 7.8), without feelings of pressure (IMI pressure/tension: mean 8.2, SD 3.7). Additionally, participants had feelings of competence (IMI perceived competence: mean 27.1, SD 5.3). They did not always feel that they could choose the exercises (IMI perceived choice: mean 19.3, SD 6.2), probably because the choice was usually made by the therapist and because some exercises have limited differentiation among several difficulty levels (eg, the participant rapidly reached the most difficult level or the step between two consecutive difficulty levels was too large).

In general, the patients with MS and therapists were positive about the DTT program. The patients perceived it as useful, challenging, interesting, and fun. They made the following statements: “it is useful, because people can train something that is related to daily life activities,” “it is challenging and interesting, because of the combination of both tasks, walking and cognition,” “more levels make the training very challenging, and when I perceived that the performances were improving, I was very satisfied,” and “the work on walking and memory is a positive aspect of the training.” The therapists also indicated that they want to keep using the system, because of “its novelty,” “the similarity to daily living,” and “the feeling that patients enjoy the training and already perceive improvements during training.” The main weak aspect, reported by both patients and therapists, was related to the too small differentiation among difficulty levels of some exercises that could make the training boring.

## Discussion

### Principal Findings

Research focused on finding new ways to administer standardized DT assessments and DTT rehabilitation interventions of high quality, make them more effective, and ensure high adherence to treatments is mandatory. For this reason, rehabilitation researchers (ie, physicians, therapists, and computer scientists) should define, design, and develop new tools that are able to assess CMI and deliver DTT. Portable and low-cost technology-based products, such as mobile phones and tablets, are the main candidates for these aims [45,46]. In fact, the benefits of adopting electronic devices instead of traditional pen and paper tools depend on several factors, such as dynamic presentation of the stimuli (eg, speed and difficulty levels according to an individual’s specific needs and progression in training), more reliable recording of cognitive and perceptual performance (eg, reaction time), standardization of the test and training environment (eg, reduced or null errors in administration), availability of faster feedback and behavioral information (eg, the time spent on each item), and reduced time in delivering and scoring exercises [37,47]. Moreover, owing to the unavoidable requirement of the execution of motor and cognitive tasks at the same time, the use of traditional computerized tools could limit or make both CMI assessment and DTT impossible. Computer-based cognitive rehabilitation systems that are usually implemented on laptops and desktop computers are not adaptable for DT exercises requiring, for example, walking or stepping on the spot, although they have been shown to be effective in improving cognitive functions [48-51]. Moreover, virtual reality or exergaming may be beneficial to improve DT performance; however, these devices are quite expensive and therefore not available in all clinical settings. Furthermore, they may not be sufficiently adaptive for people with MS having moderate-to-severe disability.

Owing to these considerations, CMI-APP, a tool based on economic, accessible, and widely-used technology (ie, tablets), was proposed to assess CMI, deliver DT exercises, and investigate DTT effects in people with MS. The tablet-based app CMI-APP implements exercises suited and tested for neurological rehabilitative interventions and is conceptualized in such a way that cognitive exercises are combinable with selected motor tasks, with presentation in an easy-to-use GUI to plan assessment and training in both cognitive and motor domains.

Here, we described the design and development of CMI-APP, as well as the results of a multicenter pilot study involving people with MS that was performed to evaluate adherence to a rehabilitation program based on CMI-APP, perceived exertion during the training, and subjective experience regarding the training.

From a technical point of view, CMI-APP provides an interactive, adaptive, user-friendly, and tailor-made interface that can help the therapist to better focus on a patient’s safety and quality of performance during DTT, without bothering about inventing new exercises, assignments, and the correctness of provided answers. Moreover, owing to these aspects of design and development, more standardized assessments of CMI and DTT and the proposal of well-designed randomized controlled trials are strongly warranted. Feedback on performance, according to the implemented cutoff values, and the variety of exercises were taken into account during the design of the app in order to improve the engagement and motivation of the patient and support the progression of the difficulty level [37]. Additionally, a clear overview of what has really been trained (ie, dosage and content) is provided through easily accessible training output logs. In addition, because several cognitive domains are trained, particular impairments in daily life may be more easily and timely identified.

The results show that this new system was very well received by patients with MS, as deduced by the high adherence to the treatment. In fact, 91% of all scheduled training sessions were completed by the patients, suggesting that this tool could be proposed for a DTT intervention in people with MS. Importantly, no adverse effects of the DTT were recorded.

On average, participants perceived the DTT as somewhat difficult. Nevertheless, participants reported that they enjoyed the training and were interested in practicing the exercises again because they considered the exercises valuable and important for preserving or improving DT performance. This result seems to be confirmed by reports recorded with an open-ended survey on training perception, revealing that training was perceived as useful, challenging, interesting, and fun. Although the patients felt that they were only relatively involved in the choice of the exercises and the difficulty level more suitable for their own abilities, they felt having competence in the execution of the proposed exercises. Probably, as a consequence, the level of stress due to the training was low, as shown by the low level of perceived pressure and tension. Although a larger sample-size study could definitively shed light on the clinical effects of CMI-APP, we are confident of the reliability of the observed usability results because our sample size matches that of previous studies on the usability of apps for MS cognitive rehabilitation [45].

Accordingly, all the therapists involved in the DTT of the 15 participants had positive feedback on CMI-APP, considered it very user friendly, and had full interest in implementing the proposed training in the clinical routine, including self-use by patients, if the aspects that they reported as weak (eg, limited differentiation among difficulty levels) were addressed in a future release of the app.

Although adherence to the treatment was very high, perception of the DTT was tolerable, and subjective experience regarding the trained activities was high, improvements in CMI-APP should be considered for use in research programs and for translation into clinical practice. In particular, it is recommended to have a wider variation in diverse assignments (eg, more words, stories, and pictures), as well as a better differentiation between the difficulty levels and an increase in the number of exercises over the diverse cognitive domains (eg, more linguistic tasks, such as verbal fluency and alternating alphabets).

CMI-APP has been recently used to assess the test-retest reliability of the 12 CMI paradigms, and the results were recently published [18]. The highest reliability was found for the motor DTC under all walking conditions (walking, walking with a cup, and walking over obstacles) in healthy controls, but the strongest was for walking alone in people with MS. Cognitive DTC appeared to not be reliable in either healthy controls or people with MS. These findings will be taken into account in future developments of CMI-APP.

Before using CMI-APP in clinical practice, large-sample studies that examine CMI and the effectiveness of a DTT intervention in people with MS, establishment of the optimal dosage per exercise, and adaptation of the thresholds for progression and regression of the difficulty level are mandatory.

Moreover, use extension to other neurological pathologies, such as stroke, Parkinson disease, and Alzheimer disease, that are shown to benefit from DTT [9-11] could be proposed and realized owing to the easy adaptability of the system to disease-dependent requirements in terms of technical aspects and exercise features.

### Limitations

There are some study limitations. First, not all the cognitive functions trainable with CMI-APP show corresponding exercises for assessment and not all motor conditions available for assessment were suggested to be adopted during the training, limiting the possibility to evaluate task-specific learning. For example, despite the use of different exercises based on vision in the training module, no visually-based exercises were included in the assessment module of CMI-APP. Even if visuospatial tests are very difficult and unsafe for execution during walking, future versions of CMI-APP should allow the execution of tasks based on vision during walking, eventually suggesting convenient set-up adaptation to preserve safety (eg, projection on the wall in front of the patient during walking on a treadmill) [52]. However, we want to clarify that the first version of CMI-APP was developed to evaluate if a carry-over general effect of training was present on CMI, and consequently, no assessment exercises specific for the training exercises were strictly required.

Second, for use in both clinical and research settings, advise to therapists will be implemented to stimulate them to train patients to walk over obstacles, carry a cup, and walk crisscross under DT conditions; moreover, the simple walking motor training condition allows the assessment of potential transfer involving more complex motor tasks (ie, walking over obstacles, carrying a cup, and walking crisscross).

Finally, to better understand the usefulness of CMI-APP and its potentiality in the market [53], a technological acceptance model [54] and client satisfaction scale [55] should be considered and administered to both patients and therapists in a study with a larger sample size.

### Conclusion

In this study, we demonstrated that CMI-APP is a tool that is safe, highly usable, motivating, and well accepted by people with MS for motor-cognitive DTT. In fact, the participants in this multicenter pilot study perceived the exercises implemented in CMI-APP as interesting, valuable, and useful to stimulate motor-cognitive abilities usually involved in daily activities. Moreover, the feeling of competence and the absence of perceived pressure are aspects that could improve the self-efficacy of people with MS [56].

Owing to these results, we are now ready for a large-sample study that examines the effectiveness of a DTT intervention with CMI-APP in people with MS, using specific clinical outcomes for motor, cognitive, and motor-cognitive performances. Moreover, despite the market presence of technological solutions providing multisensory feedback and having the ability to modulate exercise complexity, we think that positive results from a randomized controlled trial on the use of this app implementing exercises specifically suited for people with MS will suggest the most effective feedback events (eg, feedback on cadence during exercise) [37] and will play a key role in the actual exploitation of the app in the field of MS as a tool for cognitive-motor rehabilitation. However, if researchers of other neurological pathologies show interest in CMI-APP, new customized versions will be provided.

## Appendix

Multimedia Appendix 1 The “main menu” of CMI-APP. The interface allows selection of language; addition of a new patient code (entry of patient code, number of baseline steps measured in walking/stepping within one minute, and additional information of the patient) or selection of an already added patient from the drop down menu; creation of a new therapist code (entry of therapist code) or selection of an already added therapist from the drop down menu for the current session; addition of a session note for the current session (optional information); and advancement to the cognitive-motor interference assessments or the dual-task training exercises.

Multimedia Appendix 2 “Menu–Assessment” of CMI-APP usable by the therapist to select the next test for the cognitive-motor interference assessment. The performances of a patient can be assessed under a total of 19 conditions as follows: three single cognitive conditions, "DigitSpan–None," "Subtraction–None," and "Vigilance–None;" four single/dual motor conditions, "None–Cup," "None–Obstacles," "None–Crisscross," "None–Walk;" 12 dual cognitive-motor conditions, "DigitSpan–Cup," "Subtraction–Cup," "DigitSpan–Crisscross," "Subtraction–Obstacles," "Subtraction–Crisscross," "Vigilance–Crisscross," "Vigilance–Walk," "DigitSpan–Walk," "Vigilance–Obstacles," "DigitSpan–Obstacles," "Subtraction–Walk," and "Vigilance–Cup".

Multimedia Appendix 3 The cognitive task “Titrated digit span backwards” is shown. (A) Explanation of the task and the two buttons for the selection of span length determination or exercise start; (B) Interface to set the personalized span length; (C) interface for the therapist in which he/she can find the heard series of digits to repeat backwards and the numeric keypad to type what the patient responded. An answer is correct if all the numbers are correctly repeated backward.

Multimedia Appendix 4 The cognitive task “Auditory vigilance with alphabets” is shown. (A) Explanation of the task and button to start the exercise; (B) Interface for the therapist in which the current given letter is displayed and the button “YES” is present to allow the therapist to record when the patient says "yes." An incorrect answer is automatically counted as false positive (ie, a "yes" when there is no target letter) or false negative (ie, answer omission when there is the target letter). A correct answer is automatically counted in other cases (ie, a "yes" when there is the target letter and omission when there is no target letter).

Multimedia Appendix 5 The cognitive task “Serial counting backwards by 7” is shown. (A) Explanation of the task and the button to start the exercise; (B) Interface for the therapist in which the starting number is displayed and the numeric keypad to type what the patient responded is present. A correct answer is automatically displayed in green, whereas an incorrect answer is displayed in red. If the patient makes an error, he/she is requested to start again from the last correct subtraction referred.

## Abbreviations

CMI: cognitive-motor interference
DT: dual task
DTC: dual-task cost
DTT: dual-task training
EDSS: Expanded Disability Status Scale
GUI: graphical user interface
IMI: Intrinsic Motivation Inventory
MMSE: Mini-Mental State Examination
MS: multiple sclerosis
RPE: Borg 15-point Ratings of Perceived Exertion
