# Supplementary figures and images for: Design, Development, and Testing of an App for Dual-Task Assessment and Training Regarding Cognitive-Motor Interference (CMI-APP) in People With Multiple Sclerosis: Multicenter Pilot Study
Source: JMIR Mhealth Uhealth. 2020 Apr 16;8(4):e15344. doi: 10.2196/15344 (PMC7218603; doi:10.2196/15344)

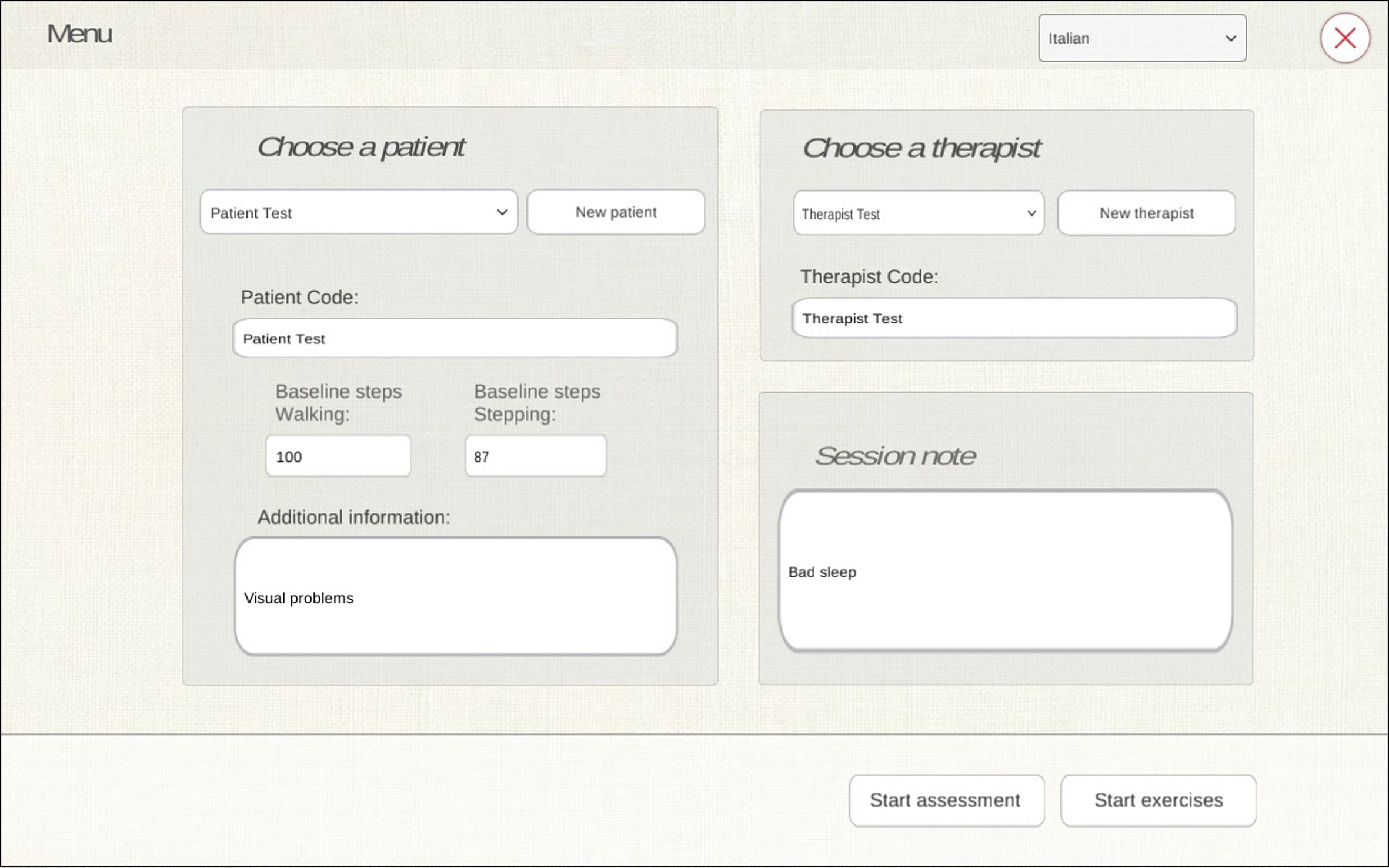

Supplement: Multimedia Appendix 1 [file mhealth_v8i4e15344_app1.png]

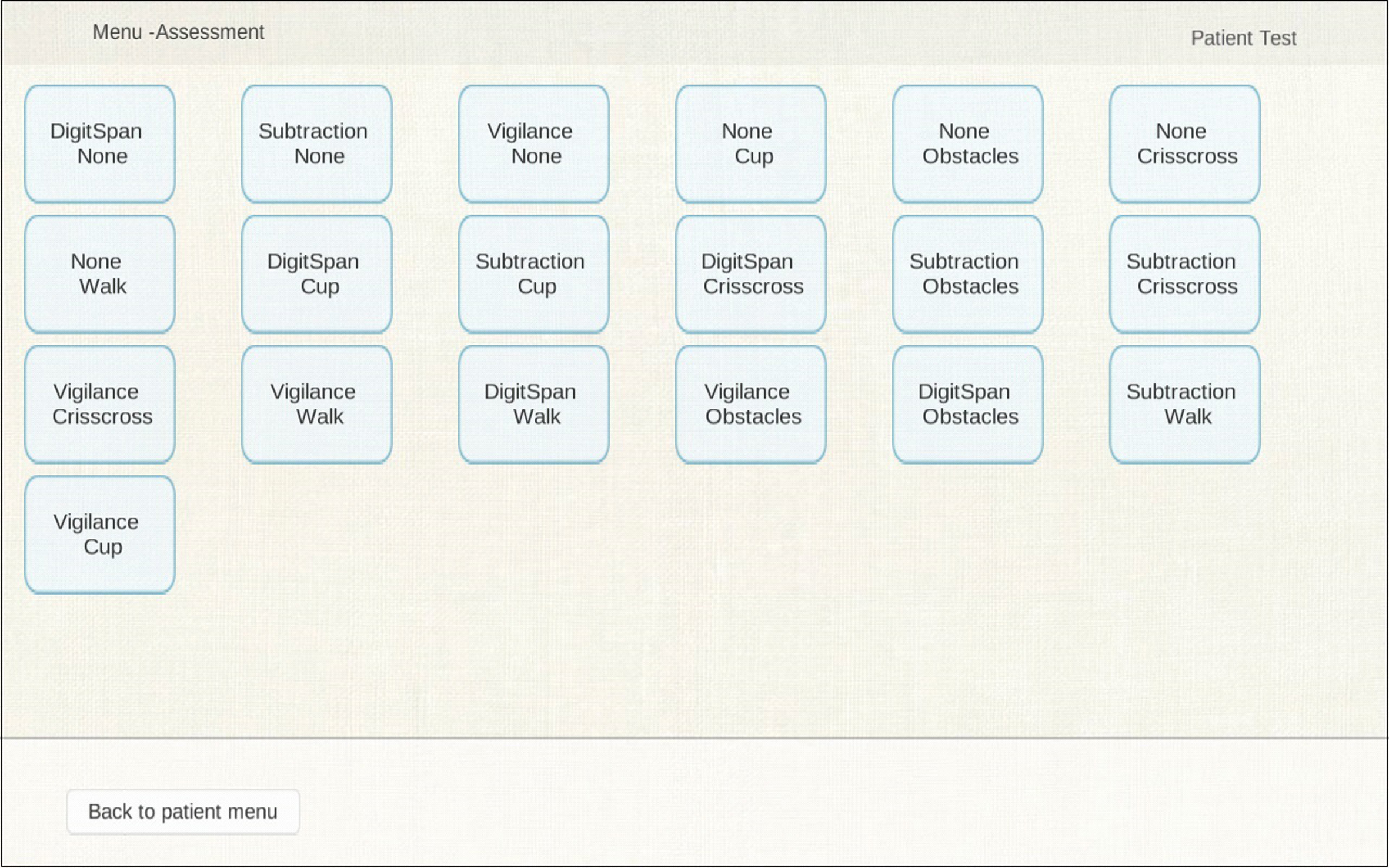

Supplement: Multimedia Appendix 2 [file mhealth_v8i4e15344_app2.png]

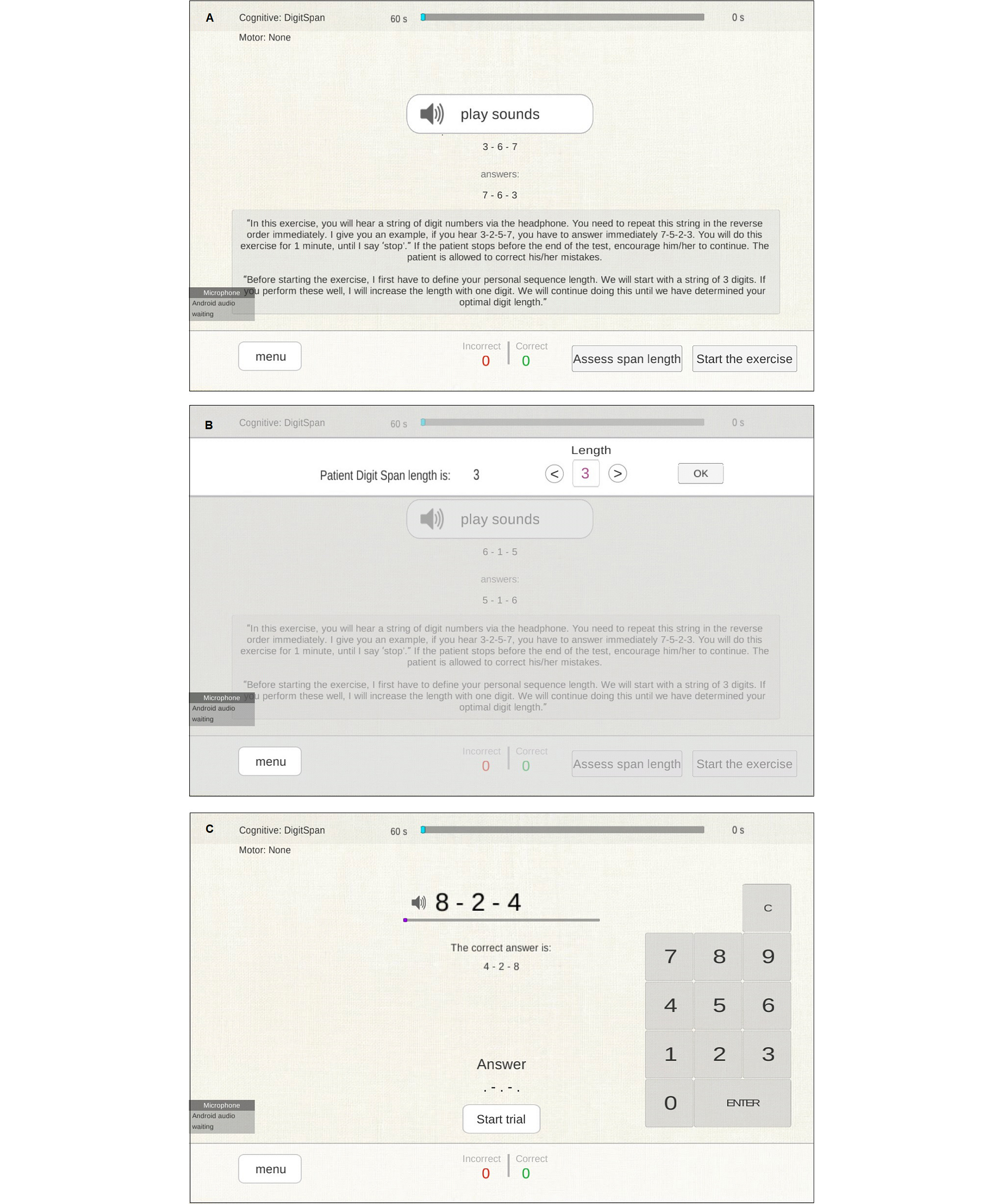

Supplement: Multimedia Appendix 3 [file mhealth_v8i4e15344_app3.png]

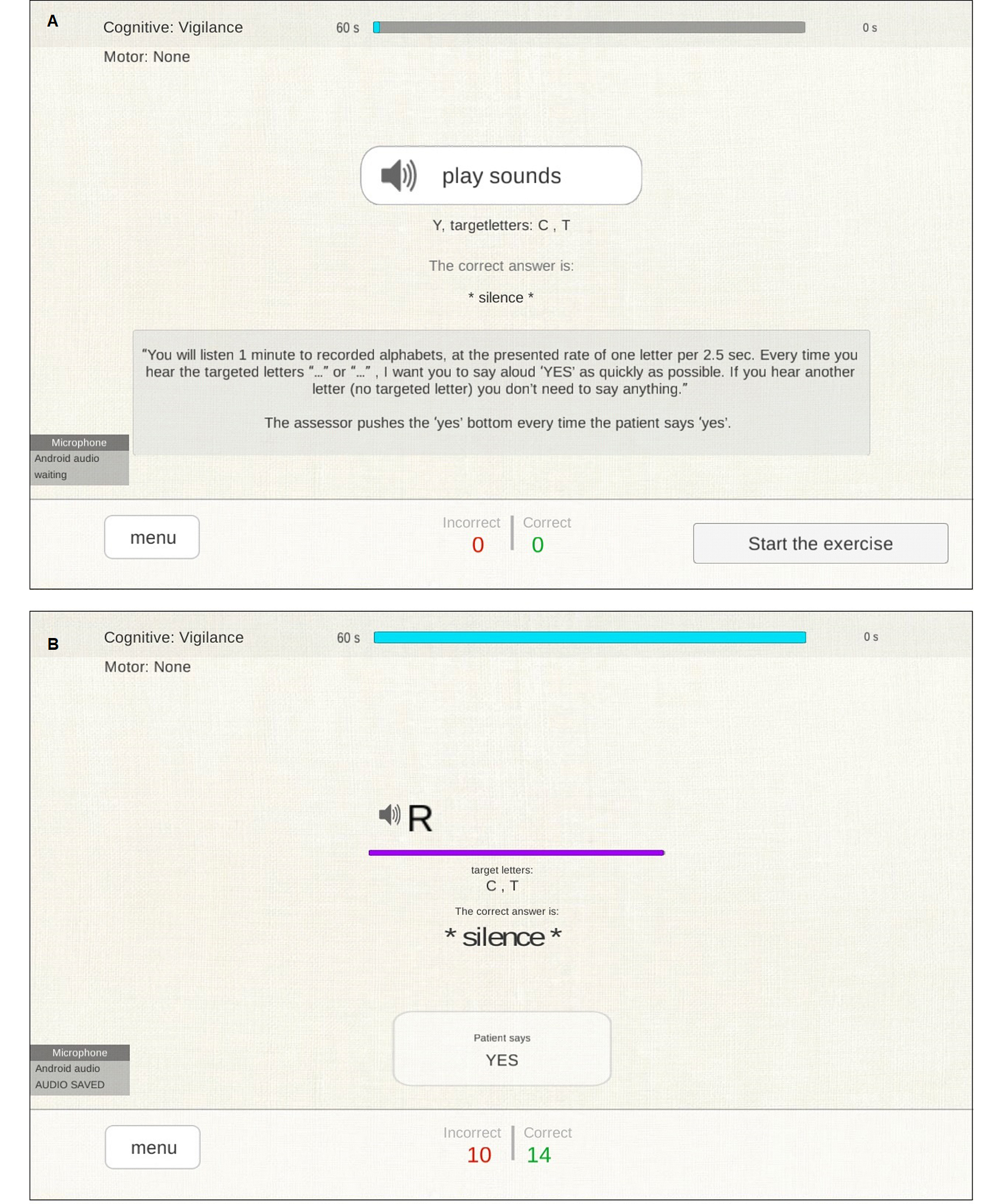

Supplement: Multimedia Appendix 4 [file mhealth_v8i4e15344_app4.png]

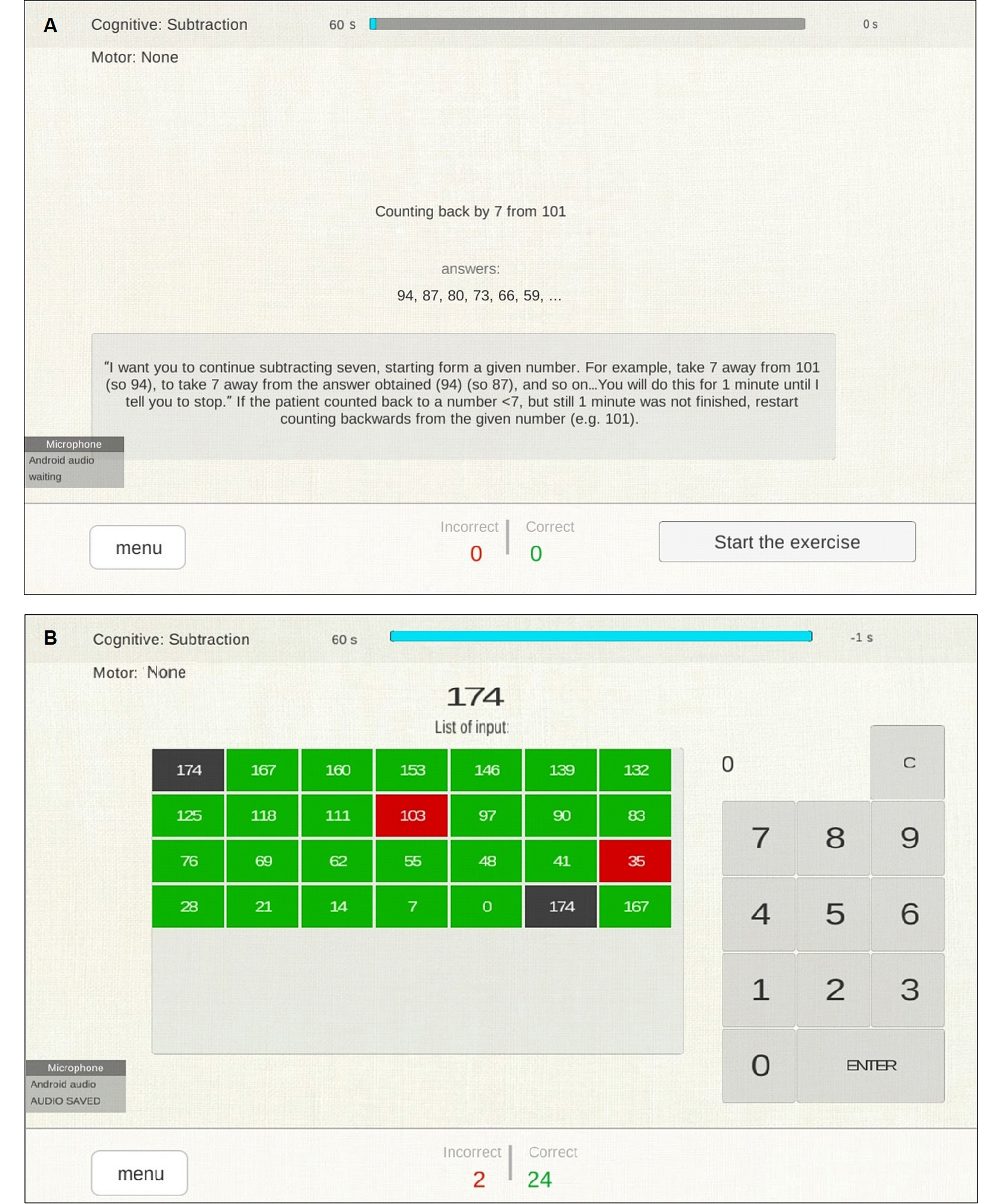

Supplement: Multimedia Appendix 5 [file mhealth_v8i4e15344_app5.png]
